# Classifying tumour infiltrating lymphocytes in oral squamous cell carcinoma histopathology using joint learning framework

**DOI:** 10.1038/s41598-025-86527-5

**Published:** 2025-01-23

**Authors:** Barun Barua, Genevieve Chyrmang, Kangkana Bora, Gazi N. Ahmed, Lopamudra Kakoti, Manob Jyoti Saikia

**Affiliations:** 1 Department of Computer Science and IT, Cotton University, Guwahati, Assam 781001 India; 2 North East Cancer Hospital and Research Institute, Jorabat, Guwahati, Assam 781023 India; 3 Dr. B Borooah Cancer Institute, Guwahati, Assam 781016 India; 4 Electrical and Computer Engineering Department, University of Memphis, Memphis, TN 38152 USA; 5 Biomedical Sensors and Systems Lab, University of Memphis, Memphis, TN 38152 USA

**Keywords:** Oral cancer detection, Biomedical engineering, Computer science

## Abstract

Oral squamous cell carcinoma (OSCC) is the most common form of oral cancer, with increasing global incidence and have poor prognosis. Tumour-infiltrating lymphocytes (TILs) are recognized as a key prognostic indicator and play a vital role in OSCC grading. However, current methods for TILs quantification are based on subjective visual assessments, leading to inter-observer variability and inconsistent diagnostic reproducibility. Only a few studies have been conducted in automating TILs quantification for OSCC, existing methods use score-based systems that focus only on tissue-level spatial analysis, overlooking essential cellular-level information and do not provide TILs infiltration subcategories required for determining OSCC grading. To address these limitations, we propose OralTILs-ViT, a novel joint representation learning framework that integrates cellular and tissue-level information. Our model employs two parallel encoders: one extracts cellular features from cellular density maps, while the other processes tissue features from H&E-stained tissue images. This dual-encoder approach enables OralTILs-ViT to capture complex tissue-cellular interactions, classifying TILs infiltration categories consistent with Broders’ grading system-“Moderate to Marked”, “Slight” and “None to Very Less.” This approach reflects pathology practices and increases TILs classification accuracy. To generate cellular density maps, we introduce TILSeg-MobileViT, a multiclass segmentation model trained using a weakly supervised method, minimizing the need for manual annotation of cellular masks and overcoming the limitations of previous TILs assessment techniques. An extensive evaluation of our methodology demonstrates that OralTILs-ViT with the configuration (Adam, $\alpha$ = 0.001) outperforms existing approaches, achieving 96.37% accuracy, 96.34% precision, 96.37% recall, and a 96.35% F1 score. Furthermore, TOPSIS analysis confirms that our method ranks first across all TILs infiltration categories. In summary, our proposed methodology outperforms single modality-representation learning approaches for accurate and automated TILs classification.

## Introduction

Oral cancer is a widespread and significant form of cancer, with Oral Squamous Cell Carcinoma (OSCC) being the most common, representing 84–97% of all cases^1^. Papua New Guinea leads globally in the incidence rate of oral cancer^2^. In South and Central Asia, oral cancer is the second most common cancer, with a five-year prevalence of 468,159, 183,566 new cases, and 103,464 deaths^2^. In India, it ranks as the second most prevalent cancer after breast cancer. Annually, India reports over 77,000 new cases and 52,000 deaths, making up a quarter of the global cases^1^. The buccal mucosa is the most frequently affected anatomical area of the oral cavity, followed by the tongue, palate, and lower lip^3,4^.

The current methods for detecting oral cancer, particularly for OSCC, involve physical examinations, imaging techniques such as MRI/CT scans, and histopathology analysis^5–7^. Of all these methods, histopathology analysis is widely recognized as the gold standard for confirming invasive cancer^8^, determining the grade of differentiation of OSCC, and providing accurate information for treatment and prognosis^9^.

In histopathological analysis, tumour lymphocytic infiltration is a key pathological feature used for grading OSCC across most grading systems^10^. Tumour-infiltrating lymphocytes (TILs), which are mononuclear immune cells that migrate into the tumour microenvironment, reflect the immune system’s response to cancer. According to the World Health Organization (WHO) recommended Broader’s grading system, TILs are classified as “Marked”, “Moderate”, “Slight” and “None” with these categories correlating to well-differentiated, moderately differentiated, and poorly differentiated tumour grades^10^ respectively. Clinical research has demonstrated the significant prognostic value of TILs in OSCC^11–14^. However, the current practice of visually quantifying TILs is subjective, leading to variability between observers and inconsistent diagnostic reproducibility. Although the International Immuno-Oncology Biomarker Working Group recently established guidelines for TILs assessment in breast cancer^15^, however, no equivalent guidelines exist for OSCC. Therefore automated method is in need of time which could offer more standardized TILs quantification and assist pathologists.

The automation of TILs quantification in OSCC has seen very limited research. Among the limited OSCC studies, two notable works are: Shaban *et al.*^13^ quantified the abundance of TILs for disease-free survival (DFS) analysis of OSCC patients. It is a tissue-level patch-based study using mostly transfer learning methods. Shaban *et al.*^14^ proposed an objective measure based on deep learning, known as the TASIL-score, to quantify tumour-associated stroma infiltrating lymphocytes in digitized images of OSCC. This study also involves tissue-level patch spatial analysis and employs a transfer learning method. The literature review reveals some limitations in existing approaches for TILs quantification in oral cancer. Current methods, such as those by Shaban *et al.*^13,14^, rely on patch-level analysis that focuses on tissue-level spatial information but neglects the critical cellular semantics needed for accurate TILs quantification. These approaches do not account for the spatial arrangement and interactions of cellular components, especially in the peritumoral stroma, which has demonstrated prognostic significance in Head&Neck squamous cell carcinoma^11,16,17^. Additionally, they lack a multiclass system and fail to explicitly quantify the spatial relationships between cells, which is vital for understanding TILs infiltration. Our study addresses these limitations by proposing a multiclass joint representation TIL classifier that integrates cellular density maps and tissue spatial relationships for a more comprehensive and accurate TILs quantification.

**Fig. 1:**
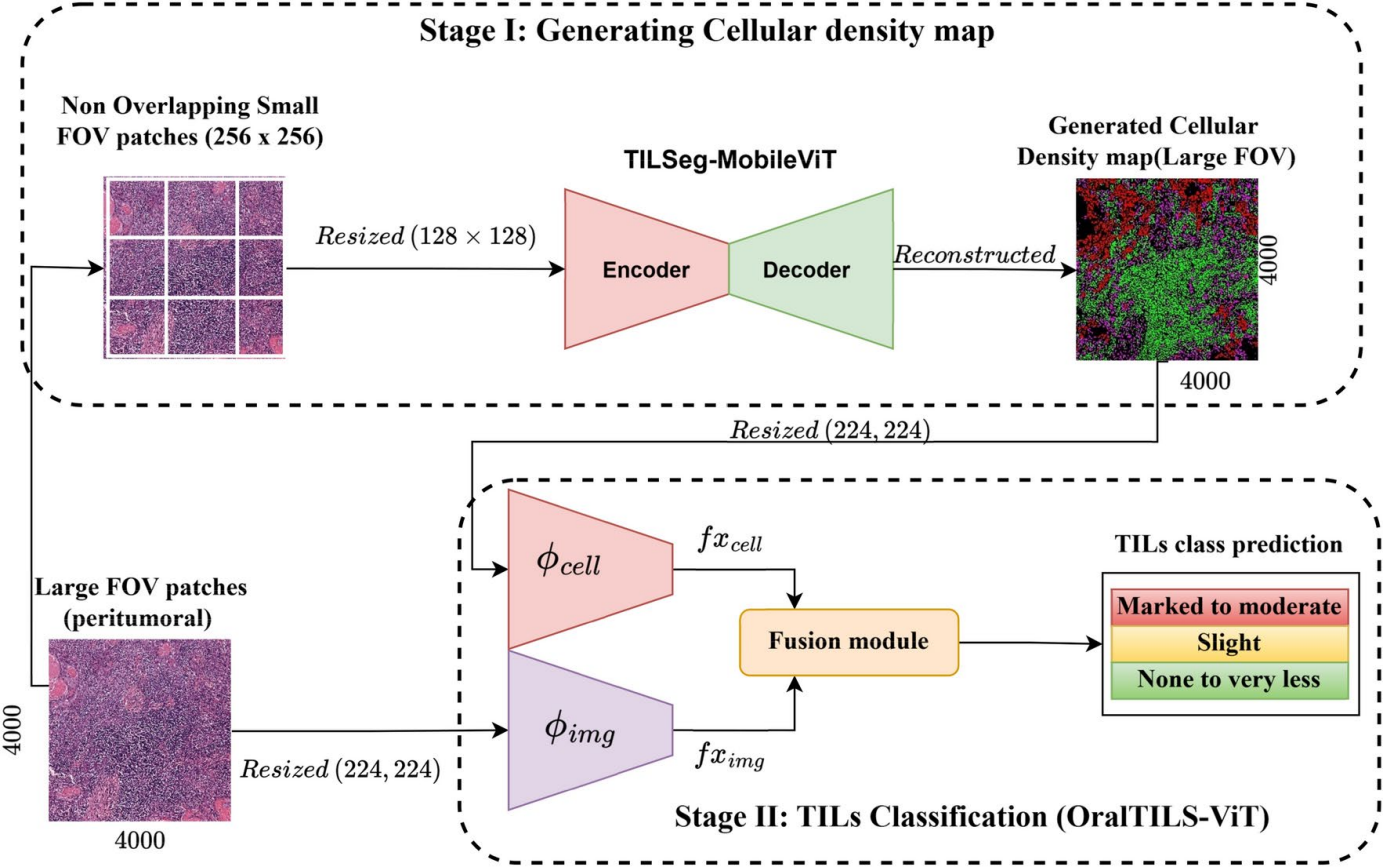
Overview of the workflow of the proposed methodology. Stage I focuses on extracting $256 \times 256$ non-overlapping small patches from larger $4000 \times 4000$ field-of-view (FOV) images, followed by resizing and generating cellular density maps using the TILSeg-MobileViT segmentation model. As part of the post-processing, the cellular density maps are reconstructed to the original $4000 \times 4000$ larger FOV by stitching the smaller patches together. Stage II classifies these larger FOV images into three TILs categories by leveraging joint embeddings from the raw H&E images and their corresponding cellular density maps.

In this paper, we introduce a novel framework for multiclass classification of TILs within the tumour microenvironment of OSCC histopathology images. Figure 1 shows the overview of the proposed framework. Our proposed framework, termed OralTILs-ViT, combines a weakly supervised cellular density map with H&E-stained tissue images to classify into three categories: *“Moderate to Marked” (moderate to significant TIL density), “Slight” (low TIL density), and “None to Very Less” (no to very low TIL density).* These classifications are based on TIL presence and follow the nomenclature from Broader’s grading system^10^. Figure 7 below illustrates the three TILs classes.

To the best of our knowledge, this study is the first to perform multiclass classification of TILs presence in OSCC using a joint representation learning method utilizing both cellular density maps and H&E-stained images. Additionally, this is the first time a next-generation CNN architecture, specifically the Mobile Vision Transformer (MobileViT)^18^, has been employed as the feature extractor. This approach addresses the limitations of existing methods by integrating detailed cellular information with tissue-level patterns, providing a more accurate classification of TILs presence in OSCC.

The major contributions of the study are summarized as follows:

- A joint representation learning framework is proposed, that integrates cellular density maps with H&E-stained patches from a larger field of view (FOV) to classify TILs in peritumoral regions of OSCC. Our method classifies TILs into three categories: “Moderate to Marked”, “Slight”, and “None to Very Less”. The framework operates in two stages.
- A novel segmentation model is introduced in stage I, TILSeg-MobileViT, which performs multiclass segmentation based on distance maps. This model generates cellular density maps for key components such as tumour cells, stromal cells, and lymphocytes, utilizing a weakly supervised learning approach.
- OralTILs-ViT, a joint representation learning classifier is introduced, which includes dual feature extractors and a fusion mechanism. This mechanism combines features from both the cellular density maps and H&E-stained images, and the fused representation is processed by a classification head to perform multiclass TILs classification.
- Conducted a comprehensive evaluation of both stages of the framework. For Stage I, we performed generalization and self-consistency tests to assess the segmentation model’s robustness. For Stage II, we compared the performance of the joint representation learning classifier against single-modality classifiers, analyzed the impact of different hyperparameter configurations, and conducted a multi-criteria decision analysis, which confirmed the superior performance and reliability of our approach.

## Methods and material

### Overview

In this study, we introduce a novel two-stage joint representation learning framework designed to classify the OSCC tumour microenvironment patches based on the density of TIL (tumour-infiltrating lymphocytes). The framework leverages both cellular density maps and H&E-stained raw RGB images to categorize TILs presence into three classes: “Moderate to Marked,” “Slight”, and “None to Very Less”. By integrating these two modalities, our framework combines their respective strengths, providing a representation that is both detailed at the cellular level and rich in contextual information, thus capturing both local cellular patterns and global tissue architecture.

The framework includes two stages: Stage I ,involves generating a cellular density map of OSCC tissue patches using multiclass semantic segmentation, focusing on key cellular components such as tumour cells, stromal cells, and lymphocytes. The rationale for creating this map is to align our approach with clinical practice, where pathologists visually assess cellular density for TIL quantification, and to incorporate important cellular spatial information into our proposed framework. This cellular information is essential for accurate TILs assessment, as it provides insights into the distribution and interaction of cells within the tumour microenvironment. However, training a supervised segmentation model requires manually annotated cells, which is expensive, labour-intensive and time-consuming. Therefore, to mitigate the dependence on manual cell annotation some previous methods^13,14^ for TIL quantification have relied on smaller field of view only tissue patch-based approach. However, these existing methods fail to capture the fine-grained cellular details that a cellular segmentation can provide. Therefore to overcome the limitations of both supervised cell segmentation and smaller field of view only tissue patch-based approaches, we employ a weakly supervised paradigm to train the proposed multiclass segmentation model. The proposed novel segmentation model is termed as TILSeg-MobileViT, inspired by the UNet architecture^19^ and Distance map based models^20^. First, it is pre-trained on a publicly available dataset. Using these pre-trained weights, we generate multiclass pseudo masks for key cellular components of OSCC tissue images. These pseudo masks are then used to fine-tune the segmentation model, enabling it to generalize effectively on OSCC tissue images and produce accurate cellular density maps.

In Stage II, we focus on classifying the OSCC H&E stained images based on TILs presence, categorizing it into three classes: “Moderate to Marked” (moderate to significant TILs density), “Slight” (low TILs density), and None to Very Less” (no to very low TILs density). To achieve this, we propose a novel architecture called OralTILs-ViT, which leverages joint representation learning of both cellular patterns and H&E-stained tissue patterns. The proposed model consists of two MobileViT^18^ modules as feature extractors referred as $\phi _{img}$ and $\phi _{cell}$, where $\phi _{img}$ is pre-trained to classify TILs presence using only H&E-stained tissue images, and $\phi _{cell}$ is pre-trained to classify TILs presence using only cellular density maps. Then the proposed fusion mechanism integrates features extracted by both $\phi _{img}$ and $\phi _{cell}$. Finally, a classification head processes these fused features to perform the TILs classification on larger field of view images.

The Fig. 1 illustrates the overall architecture and workflow of the proposed framework.

### Datasets

- *PanopTILs* is a comprehensive large-scale dataset designed for detailed analysis of the tumour microenvironment (TME) in whole-slide images (WSIs) of H&E-stained breast cancer slides^17^. It features annotated boundaries of histologic regions, along with the locations, classification labels, and segmentation boundaries of nuclei within the same fields of view. PanopTILs was developed by integrating and expanding two existing public datasets, BCSS^21^ and NuCLS^22^. The dataset encompasses data from 151 patients across various hospitals, including 1709 regions of interest and 814,886 annotated nuclei (annotations are mostly bootstrapped using a pre-trained model). This dataset is used in Stage I to pre-train the segmentation model, primarily to capture the generic spatial context of cellular structures. Its extensive annotations, which provide a rich representation of cellular distributions, align well with the objectives of our study. This makes the dataset highly suitable for transfer learning across different cancer types, including OSCC, as demonstrated in previous segmentation work^23–25^ that leveraged datasets from other domains.
- *TCGA-HNSCC* is a public dataset comprised of 560 whole-slide images (WSIs) focused on head and neck cancer histopathology. From this collection, we selected 146 WSIs specifically related to OSCC. These WSIs cover various sites, including the floor of the mouth (56), lips (3), gums (11), palates (5), and other ill-defined regions within the lip and oral cavity (71). This subset is named *OSCC* $_{tcga}$ which will be used in this study.

The datasets were used in different settings for each stage’s training and validation, which discussed in detailed in following sections.

### Stage I: generating cellular density map

As discussed above, the cellular spatial information is important in classifying TILs. Therefore, this stage focuses on generating a cellular density map that provides spatial arrangement information of key cellular components. To achieve this, we introduce a multiclass segmentation model named *“TILSeg-MobileViT”*, designed to segment essential cellular components-tumour cells, stromal cells, and lymphocytes. The proposed model is trained using a weakly supervised approach due to the limitations of fully supervised methods, which require manually annotated cellular masks-a process that is expensive, time-consuming, and labour-intensive. To address this challenge, we first identified an existing dataset, PanopTILs^17^, with cellular morphology closely aligned with OSCC, which was adapted to meet the needs of this study. This dataset was used to pretrain the model, enabling the generation of pseudo masks. After pretraining, the model was fine-tuned using OSCC tissue patches with the generated pseudo masks. The resulting output from this stage is an RGB multiclass cellular density map, as depicted in the Fig. 7. This serves as one of the inputs for subsequent downstream tasks, providing comprehensive information on the distribution of cellular components within the tissue. Further details of this stage are discussed in the following sections.

#### Dataset setting for stage I

***For pretraining:*** To pretrain our proposed model, we utilized the PanopTILs dataset^17^. However, the initial format of PanopTILs required several modifications before it could be effectively used for pretraining. In the PanopTILs dataset, each mask is provided as an ($m$ , $n$ , 3) PNG image, where $m$ and $n$ denote the dimensions of the region of interest (ROI), and the three channels represent:

- First Channel: Region semantic segmentation mask
- Second Channel: Nucleus semantic segmentation mask
- Third Channel: Binary mask of nuclear boundary edges

For our specific problem, we focused on the second channel, which contains the nucleus semantic segmentation mask. We extracted this channel and discarded the others. However, the original labelling in this channel did not match our requirements, so we relabeled the second channel as follows: *0 for Background, 1 for tumour (cancer nucleus), 2 for TILs (Lymphocyte nucleus + Plasma cell/large TIL nucleus), 3 for Stroma (Stromal nucleus + Large stromal nucleus), and 4 for Others (Other nucleus, Unknown/Ambiguous nucleus).*

In addition, our model requires distance maps as auxiliary information for segmentation tasks. The rationale behind this is that distance maps help differentiate overlapping cells during segmentation as shown in some previous studies^23,24^. To generate these distance maps, we first convert the segmentation mask into a binary mask, where the cells are labelled as ‘1’ (white) and the background as ‘0’ (black). Then apply the Euclidean distance transform algorithm to the binary mask. This transform calculates the shortest distance from each background pixel to the nearest cell boundary, producing a distance map where pixels near cell boundaries have lower values and those farther away have higher values.

After that to compile the dataset for this stage pretraining, we extracted non-overlapping patches of size $256\times 256\times 3$ from 1709 regions of interest (ROIs), each originally $1024\times 1024\times 3$ in dimension. This extraction process yielded approximately 27,344 patches. After filtering out unwanted patches, the final dataset consisted of 25,594 patches, including raw images, generated multiclass semantic masks, and generated distance maps. This dataset was then split into training (80%), validation (10%), and test (10%) subsets. All images are resized to $128\times 128\times 3$ for segmentation.

***For finetuning:*** To fine-tune our proposed model, we created patches of size $256\times 256\times 3$ from the *OSCC* $_{tcga}$ dataset. We selected approximately 45,600 patches where cellular components were most prevalent. Using the proposed segmentation model with pre-trained weights, we generated pseudo-segmentation masks for these patches. To ensure high confidence in the cellular components identified, the segmentation threshold was set to greater than 0.95. After filtering unwanted patches, we compiled a fine-tuning dataset consisting of 45,454 patches each consisting of OSCC tissue patches, multiclass pseudo-masks, and distance maps. This dataset was then divided into training (80%), validation (10%), and test (10%)subsets. All images are resized to $128\times 128\times 3$ for segmentation.

#### Proposed segmentation model architecture for stage I

We proposed TILSeg-MobileViT, a multiclass segmentation model inspired by distance-based nuclei segmentation models^20,23^ and popular architectures like UNet^19^ and Attention-UNet^26^. Distance-based models are highly effective in separating dense and overlapping cells, a key challenge in segmentation tasks. These models are often based on UNet^19^ type architectures because of their ability to capture deep feature representation and focus on relevant areas through skip connections during upsampling, which enhances the segmentation of biomedical images. However, such models typically involve a large number of parameters and rely on complex multi-branch designs, increasing computational complexity.

To address these limitations and align with our study objectives, we proposed a lightweight segmentation model that uses a transformer-based encoder and adopts a multi-head output design instead of a multi-branch approach with total parameter of 10,497,540. Our architecture, comprising an ***Encoder, skip connections, attention gates, and a multihead decoder***, efficiently balances performance and complexity by downsampling input images, extracting deep feature representations, and generating segmentation masks through upsampling.

**Fig. 2:**
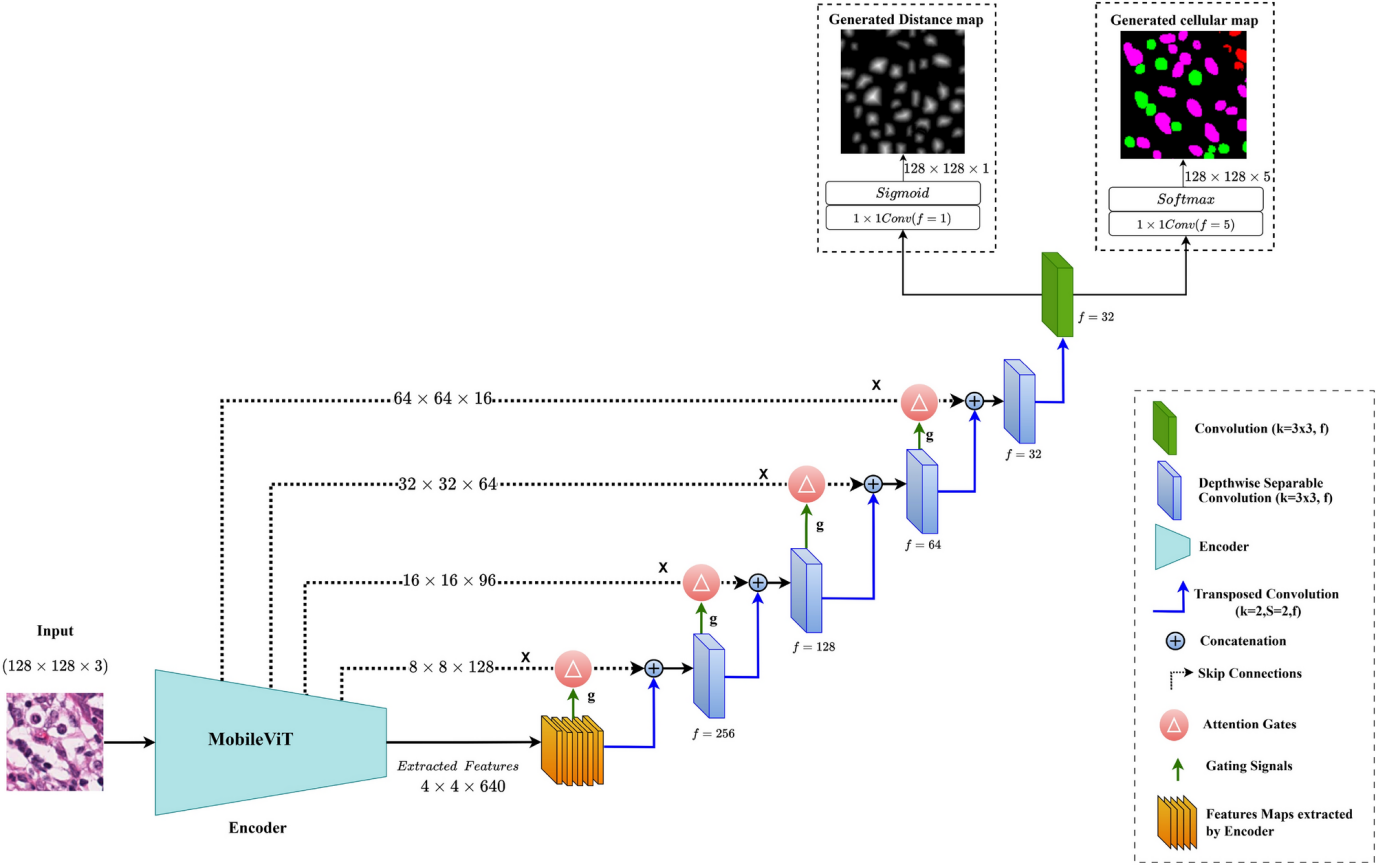
Architecture of proposed segmentation model (TILSeg-MobileViT) for generating cellular density map.

Our architecture uses *“MobileViT”* as an encoder. MobileViT was proposed by Mehta et al.^18^, a lightweight and general-purpose vision transformer for mobile devices. It represents a hybrid model that merges vision transformers with convolutional layers, designed to leverage the strengths of both approaches i.e. to capture both local and capture long-dependencies and global contextual spatial information. This dual capability proves to help in semantic segmentation tasks. To the best of our knowledge, this is the first work which employs MobileViT^18^ as an encoder in a distance map UNet inspired segmentation model in a histopathology setting. The encoder accepts an input tensor of size $128\times 128\times 3$.

Additionally, as shown in Fig. 2 we have also introduced attention gates as Attention-UNet^26^ with skip connections to reintroduce relevant information from the encoder pathway to the decoder pathway which also helps in increasing the accuracy of localizing foreground pixels.

As shown in Fig. 2 the decoder of our proposed model follows the following sequence at each stage—Firstly it performs up sampling operation that increases the spatial resolution of the feature maps. These upsampling operation is implemented through 3$\times$3 transposed convolution (deconvolution), after that skip connection from the corresponding encoder pathway is passed through attention gates, and this attention modulated information is concatenated with an upscaled feature map. Subsequent to concatenation decoder performs 3$\times$3 depth-wise separable convolution followed by ReLU activation. The depthwise separable convolution is made up of 3$\times$3 depth-wise convolution and 1$\times$1 pointwise convolution along with batch-normalization and ReLu. The rationale behind using depth-wise separable convolution instead of standard convolution is that depthwise is more efficient in terms of computational parameters which help to reduce the total trainable parameter of our model.

The architecture’s final layer includes two distinct output heads: *an auxiliary head* and a *main head*. Each head serves a specific purpose in the task of cellular component segmentation.

- The auxiliary head is designed to generate distance maps, which provide valuable spatial information that aids in differentiating between closely situated cellular components. This head consists of a convolutional layer with a kernel size of 1$\times$1 and a single filter, followed by a sigmoid activation function. The output of this head is a distance map where each pixel represents the distance to the nearest boundary of a cellular component. This distance information is crucial for distinguishing between adjacent cells and for delineating the boundaries more accurately^20,23^
- The main head focuses on generating the multiclass segmentation mask for cellular components. It consists of a convolutional layer with a kernel size of $1\times 1$ and five filters, followed by a SoftMax activation function. Each filter corresponds to one of the five classes: background pixels, tumour cell pixels, stroma cell pixels, lymphocyte cell pixels, and other irrelevant pixels. The SoftMax activation function ensures that the output values represent class probabilities, facilitating the assignment of each pixel to one of the predefined classes.

Both the *auxiliary* and *main head* are trained simultaneously, leveraging a combined loss function that incorporates contributions from both outputs. The auxiliary head’s distance maps serve as a supplementary signal that improves the segmentation performance of the main head. By generating distance maps, the auxiliary head provides additional spatial cues that help the model learn to handle overlapping or closely spaced cells. During training, the gradients from the auxiliary head’s loss function contribute to the learning process of the main head, encouraging it to produce non-overlapping and more precise cellular boundaries^20,23^. This complementary information helps the main head in refining the segmentation masks, particularly in areas where cells are in close proximity or overlap.

The loss functions used during the training of this stage are as follows:

- For the *main segmentation head*, the loss functions are Focal Loss^27^ and Dice Loss^28^. Focal Loss is effective for distinguishing between majority and minority classes, while Dice Loss helps in refining the segmentation output, improving the shape and smoothness of the segmented regions. Combining these two losses ensures that the model optimizes not only for pixel-wise classification but also for global region-level accuracy.
- For the *auxiliary head*, the loss function used is Mean Squared Error (MSE) Loss.

The mathematical representation of the total loss function is as follows:

1 $$\begin{aligned} {\mathscr {L}}_{\text {Total}} = \lambda _1 \cdot {\mathscr {L}}_{\text {Auxiliary}} + \lambda _2 \cdot {\mathscr {L}}_{\text {Main}} \end{aligned}$$

where:

- ${\mathscr {L}}_{\text {Total}}$ is the total loss,
- ${\mathscr {L}}_{\text {Auxiliary}}$ represents the loss function for the auxiliary head (MSE Loss),
- ${\mathscr {L}}_{\text {Main}}$ represents the combined loss function for the main head, which includes both Focal Loss and Dice Loss,
- $\lambda _1$ and $\lambda _2$ are the weights (scalars) for each of the losses to balance their contributions. These weights can be set or tuned during training; in this case, we assign equal weights to $\lambda _1$ and $\lambda _2$, both set to 1.

After that, each class label corresponding to a cell type is assigned a colour in the RGB colour model to create the cellular density map. Tumour cells are marked in red, TILs are marked in green, and stromal cells are marked in dark pink as shown in Figs. 4 and 7.

### Stage II: TILs classification

In this stage, we propose a novel joint representation learning classifier for the classification of tumour microenvironment patches into three categories: “Moderate to Marked” , “Slight”, and “None to Very Less”. The proposed framework introduces a dual-branch convolutional neural network architecture, termed OralTILs-ViT, which leverages joint representations of cellular density maps and raw hematoxylin and eosin (H&E) stained images as shown in Fig. 1. The Architecture and Joint Representation rationale is that the OralTILs-ViT architecture integrates two complementary data modalities: cellular density maps, generated from the initial segmentation stage, and the raw H&E stained images. The cellular density maps provide fine-grained information about cellular distribution and patterns, highlighting the spatial localization and density of relevant cellular components. This detailed representation is crucial for capturing the intricate patterns of TILs within the tissue microenvironment. Conversely, raw H&E-stained images offer contextual information of tissue and capture the broader tissue architecture and provide crucial contextual information.

However, while cellular density maps excel in presenting cellular patterns, they lack broader contextual information about the tissue regions and raw images often lack the ability to provide fine-grained information of cellular structures therefore by combining these two modalities, the proposed OralTILs-ViT model creates a joint representation that capitalizes on the strengths of both. The synergy between the two modalities ensures that the learned representation is not only detailed at the cellular level but also contextually rich, capturing both local cellular patterns and global tissue architecture. This comprehensive representation is empirically validated in our results section, where we demonstrate that the dual-branch approach leads to superior classification performance compared to using only one modality alone.

#### Dataset setting of stage II

The dataset for Stage II comprises two types of images—Raw H&E-stained tumour microenvironment (TME) patches with a larger field of view and generated corresponding cellular density maps To create the dataset, 40 WSIs were selected from the *OSCC*$_{tcga}$ dataset. The selection process was guided by two pathologists on our team. From these WSIs, we generated a pool of 9430 non-overlapping patches, each sized 4000 $\times$ 4000 pixels. These larger field-of-view patches were created using a sliding window tiling technique, with specific criteria to maximize tissue content:

- Only patches with more than 50% tissue coverage were selected to avoid blank areas and artifacts.
- Preference was given to patches with a high composition of cellular (hematoxylin-rich) and acellular (eosin-rich) regions. This favoured tiles with more peritumoural stroma^17^. Peritumoral stoma areas are more informative for TILs infiltrations^15^.

The tiling process was performed using the HistoLab library^29^. Rationale for choosing larger patches is that previous deep learning-based TILs infiltration studies rely on smaller field of view patches. However, smaller field of view patches limit the model’s ability to capture broader architectural relationships and cellular interactions within the TME, which is important for TILs quantification . By using larger $4000\times 4000$ field of view patches, our approach considers the inclusion of broader spatial context, enabling the model to analyse the heterogeneous tumour microenvironment and a more comprehensive representation of cellular and acellular regions, facilitating the identification of long-range cellular interactions and broader tissue architecture. From the pool of 9430 unlabelled patches, 4054 patches were manually labeled into three classes based on TILs infiltration levels—“Moderate to Marked”, “Slight” and “None to Very Less”. A corresponding cellular density map for each labeled patch was then generated using the segmentation model developed in Stage I. This resulted in a paired dataset of raw patches and their corresponding cellular density maps, which was used to train the proposed joint representation classifier. The dataset was split into training and validation sets in an 90:10 ratio. To address class imbalance during training, we applied a class weighting technique. Additionally, data augmentation techniques, including random rotation (horizontal and vertical), brightness adjustment, and stain color augmentation, were employed to enhance the size and diversity of the training set. A separate labeled test set was curated from the remaining unlabeled patches, containing the class distributions as None to very less—520, Slight—516 and Marked to moderate—454. To ensure computational feasibility during training, the paired raw images and cellular density maps were downscale to $224\times 224$. Anticipating that this downscaling could result in the loss of critical spatial context from the larger field of view, therefore we proposed the joint representation learning framework to leverages the complementary cellular density maps to preserve the broader spatial context, enabling the model to retain and effectively analyze key interactions and features within the larger tumour microenvironment (TME) for TILs classification.

#### Proposed architecture of TILs classifier

**Fig. 3:**
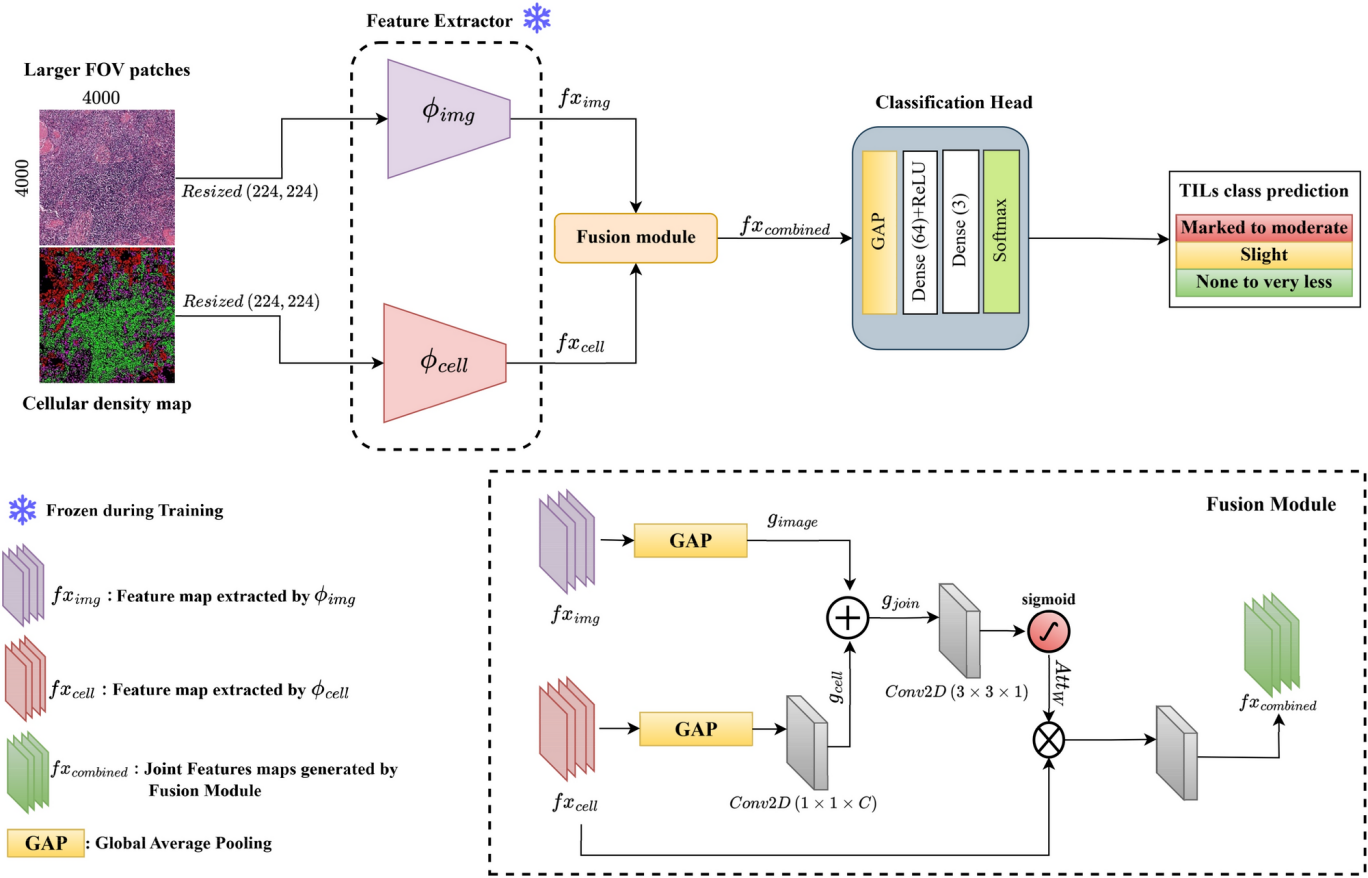
Architecture of proposed TILs classifier—OralTILs-ViT, which comprises of two feature extractors ($\phi _{img}$ and $\phi _{cell}$), a Fusion module and a Classification head.

OralTILs-ViT is the proposed architecture that performs the classification of TILs. The detailed architecture is showcased in the Fig. 3. The main components of the proposed model are two feature extractors ($\phi _{img}$ and $\phi _{cell}$), a Fusion module and a Classification head. The details of each component are discussed below:

- *Feature extractor:* Proposed model have two feature extractor termed as $\phi _{img}$ and $\phi _{cell}$, both the extractor use MobileVit^18^ as encoder, but pretrained differently. $\phi _{img}$ is responsible for extracting features from raw images, it accepts raw images of dimension $224\times 224\times 3$. It was pretrained to perform classification based only on raw images. And $\phi _{cell}$ is responsible for extracting important features from the cellular density map therefore it was pretrained to solve the classification of TILs using only the cellular density map. During pretraining both the encoder learn to extract deep important representations.
- *Fusion module:* From Fig. 3 of the fusion module, we observe that the features $fx_{cell}$ and $fx_{image}$, which are extracted from the encoder, are passed to a fusion module to obtain a joint representation. As illustrated in Fig. 3, the initial operation performed on these two features is Global Average Pooling ($GobalAvg_{Pooling}$), which creates two global representations, $g_{cell}$ and $g_{image}$. To obtain the global joint representation $g_{join}$, we perform element-wise summation ($\oplus$) between $g_{cell}$ and $g_{image}$. However, before the summation, $g_{cell}$ is fed through a $1 \times 1$ Conv2D layer to transform it, making it suitable for element-wise summation and ensuring it has a similar channel dimension as $g_{image}$. Next, to obtain the attention weights $Att_{W}$, $g_{join}$ is fed into a $3 \times 3$ convolution layer followed by a sigmoid activation function ($\sigma$). These attention weights $Att_{W}$ are then used to modulate $fx_{cell}$ by performing element-wise multiplication followed by $1 \times 1$ convolution layer with 256 filters along with batch normalization, resulting in a joint feature map $fx_{combined}$. This joint feature map $fx_{combined}$, which incorporates cellular density and tissue information passed to the classification head.
- *Classification head:* The classification head transforms joint features into class probabilities through a series of operations. It starts with GlobalAveragePooling2D, reducing spatial dimensions, followed by a Dense(64) with 64 units for feature compression and a dropout is utilized as a regularizer. Finally, the output of the dense layer is passed through the final output dense layer with 3 units and a softmax activation function, denoted as Dense(3, softmax), which computes the class probabilities for classification.

The loss function used in training for the classifier is the cross-entropy loss. The formula for cross-entropy loss is given by:

2 $$\begin{aligned} {\mathscr {L}}_{\text {CE}} = - \frac{1}{N} \sum _{i=1}^{N} \left( y_i \log (p_i) + (1 - y_i) \log (1 - p_i) \right) \end{aligned}$$

where:

- $N$ is the total number of samples,
- $y_i$ is the ground truth label for sample $i$,
- $p_i$ is the predicted probability for sample $i$,
- $\log$ denotes the natural logarithm.

## Training and implementation details

All the experiment are conducted using one Nvidia RTX 3060 6GB GPU. All the training implementations are done using the open-source package Keras and TensorFlow. Other packages used for image processing and analysis are Numpy, pandas, Opencv, and scikit-learn.

The training and implementation of the models were conducted in two distinct stages, each carefully designed to optimize performance on the respective tasks. In *Stage I*, the segmentation model, TILSeg-MobileViT, was initially trained on the PanopTILs dataset. The training employed a combination of Focal Loss^27^ and Dice Loss^28^ for the main segmentation head, while the distance output head utilized Mean Squared Error (MSE)^30^ as the loss function. The entire model was trainable, and the training used the Adam optimizer^31^ with a learning rate of $\alpha$ = 0.001 and a batch size of 8. Early stopping with a patience of 8 epochs was applied, and the model converged after 30 epochs.Subsequently, the model was fine-tuned on the *OSCC*$_{tcga}$ dataset. During fine-tuning, all layers were frozen except for the last 20 layers, which were unfrozen for further training. The same loss functions were employed, and the Adam optimizer with a learning rate of $\alpha$ = 0.001 and a batch size of 16 was used. Fine-tuning concluded after 20 epochs, ensuring the model adapted effectively to the oral cancer domain.

*Stage II* focused on training the OralTILs-ViT model, which combines features from raw images and cellular density maps. This stage involved pretraining two feature extractors Only Image ($\phi _{img}$) and Only Cell density map ($\phi _{cell}$), along with proposed classification heads followed by the training of OralTILs-ViT. The feature extractor $\phi _{img}$ was pretrained on raw images from the *OSCC*$_{tcga}$ dataset. The only cellular density map feature extractor ($\phi _{cell}$) was pretrained on cellular density maps from the *OSCC*$_{tcga}$ dataset generated using Stage I. After pretraining, the fusion module and classification head of OralTILs-ViT were trained on combined features (raw images + cellular density maps). During training, the feature extractors $\phi _{img}$ and $\phi _{cell}$ were frozen, and cross-entropy was used as the loss function. Various hyperparameter settings were explored to evaluate their impact on the performance, the combination of parameters explored are Learning rates of $\alpha$ = 0.001 and 0.0001 were tested with both the Adam and SGD optimizers, resulting in convergence within 6–30 epochs.

All the training details and hyperparameter settings is summarized in Table 1.

**Table 1:** Summary of hyperparameters and training details for Stage I and Stage II models.

Stage	Model	Dataset	Loss functions	Batch size	Optimizer	Learning rate $\alpha$	Convergence epochs
Stage I (Pre-training)	TILSeg-MobileViT	PanopTILs	Focal+Dice(Main Head), MSE (Distance Head)	8	Adam	0.001	30
Stage I (Fine-Tuning)	TILSeg-MobileViT	*OSCC*$_{tcga}$	Focal+Dice(Main Head), MSE (Distance Head)	16	Adam	0.001	20
Stage II	Only Image($\phi _{img}$)	Raw Images (*OSCC*$_{tcga}$)	Cross-Entropy	16	Adam	0.001	16
						0.0001	6
					SGD	0.001	9
						0.0001	30
Stage II	Only Cell density map($\phi _{cell}$)	Cellular Density Maps (*OSCC*$_{tcga}$)	Cross-Entropy	16	Adam	0.001	12
						0.0001	8
					SGD	0.001	10
						0.0001	8
Stage II	OralTILs-ViT	Combined Features (Raw Images + Cellular Density Maps *OSCC*$_{tcga}$)	Cross-Entropy	16	Adam	0.001	10
						0.0001	7
					SGD	0.001	8
						0.0001	10

## Results

### Segmentation performance evaluation

The proposed segmentation model’s performance is compared with two popular segmentation methods, UNet and Attention-UNet, as no other segmentation methods have been previously applied to the mentioned dataset. We chose to compare our model with these two methods because they are widely recognized and established benchmarks in the field of medical image segmentation. Additionally, our primary objective in this study is TILs classification with joint representation learning, rather than an extensive comparison of segmentation models. Therefore, limiting the comparison allows us to focus on the main objective while still demonstrating the segmentation efficacy of our model. In the future, this comparison can be extended to include other segmentation models as a separate study. The metrics used for this evaluation are Intersection Over Union (IoU) and Dice score for each cellular type i.e. tumour, stroma and TILs. IoU can be calculated as (3) and Dice score can be calculated as (4)

3 $$\begin{aligned} \text {IoU} = \frac{|A_{\text {predicted}} \cap A_{\text {ground truth}}|}{|A_{\text {predicted}} \cup A_{\text {ground truth}}|} \end{aligned}$$

4 $$\begin{aligned} \text {Dice} = \frac{2 \times |A_{\text {predicted}} \cap A_{\text {ground truth}}|}{|A_{\text {predicted}}| + |A_{\text {ground truth}}|} \end{aligned}$$

where $A_{predicted}$ represents the set of pixels in the predicted segmentation and $A_{ground truth}$ represents the set of pixels in the ground truth segmentation.

**Table 2:** Performance analysis of proposed segmentation model of each cell type on test set (PanopTILs dataset). Significant values are in bold.

Cell type	Methods	IoU	Dice
Tumours	UNet	0.4959	0.5694
	Attention-UNet	0.4749	0.5492
	**Proposed**	**0.6955**	**0.7603**
TILs	UNet	0.5834	0.6407
	Attention-UNet	0.6043	0.6591
	**Proposed**	**0.6483**	**0.7048**
Stroma	UNet	0.3642	0.4539
	Attention-UNet	0.3800	0.4679
	**Proposed**	**0.5150**	**0.5992**
Average of all cell type	UNet	0.4812	0.5547
	Attention-UNet	0.4864	0.5587
	**Proposed**	**0.6196**	**0.6881**

From Table 2 it is observed that the proposed segmentation method i.e TILSeg-MobileViT, demonstrates superior performance compared to UNet and Attention-UNet across all cell types (Tumours, TILs, and Stroma) based on IoU and Dice scores. For tumour cell types, the proposed model achieves significantly higher scores (IoU: 0.6955, Dice: 0.7603) compared to UNet and Attention-UNet, indicating better segmentation accuracy. In TILs segmentation, the proposed model also outperforms the others, with an IoU (0.6483) and a Dice score (0.7048), showing improved efficacy over both UNet and Attention-UNet. For stroma cells, which are challenging to segment, the proposed model again excels with higher IoU (0.5150) and Dice score (0.5992), compared to the lower performance of UNet and Attention-UNet. Overall, the proposed model consistently delivers better results across all cell types, with an average IoU (0.6196) and Dice score (0.6881), underscoring its effectiveness in the segmentation of relevant cell types.

**Fig. 4:**
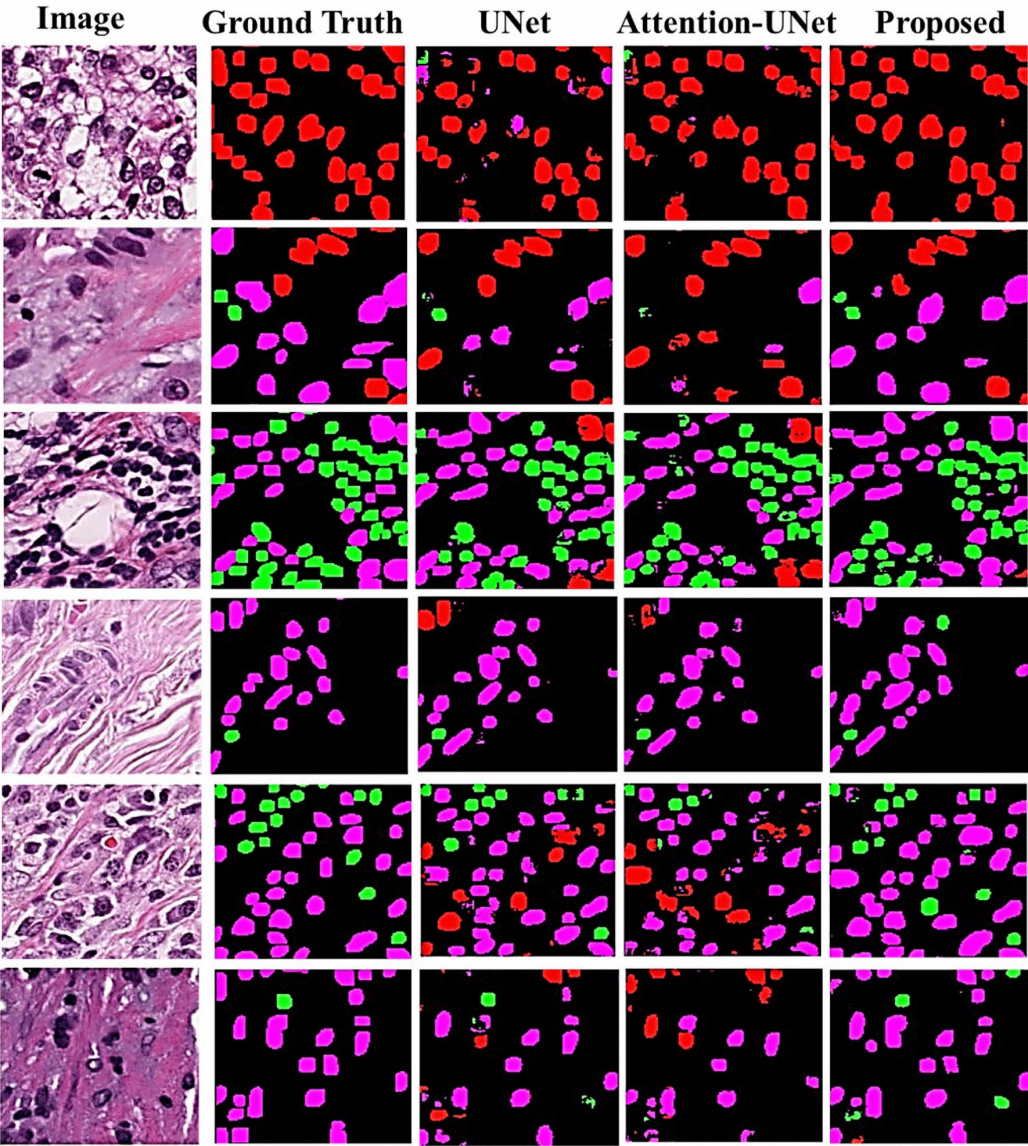
Examples of results generated by compared models on test set of PanopTILs dataset. (Red color represents Tumour cells, Green color represents Lymphocyte/plasma cell, Dark pink color represents Stroma cells).

Figure 4 presents examples of segmentation masks generated by the proposed method alongside those produced by other methods. Qualitatively, it is evident that the masks generated by the proposed method are more accurate. Specifically, the proposed method’s masks show a higher degree of overlap with the Ground Truth. For instance, in the first image of the figure, the Ground Truth mask contains all tumour cells. However, both UNet and Attention-UNet fail to preserve the cell structure and misclassify some cells. In contrast, our proposed method correctly classifies all cells as tumour cells and preserves their structure better compared to the other methods.

### Generalization evaluation of proposed segmentation model

To evaluate the generalization capability of our proposed segmentation model on the *OSCC*$_{tcga}$ dataset, as it is not trained on this dataset. As ground truth masks are unavailable, we devised an indirect validation/generalization evaluation test. This test is based on the principle that certain cell types predominate in specific tissue regions: for example, tumour tissue regions primarily contain tumour cells, stroma tissue regions predominantly feature stroma or connective cells, and lymphocyte regions mainly consist of lymphocytes or plasma cells. The rationale behind this test is to validate the model’s segmentation and generalization capability by leveraging expected cellular distributions within different tissue types without any semantic ground truth masks. This approach allows us to indirectly evaluate the model’s performance based on the natural cellular composition of the tissue regions.

**Fig. 5:**
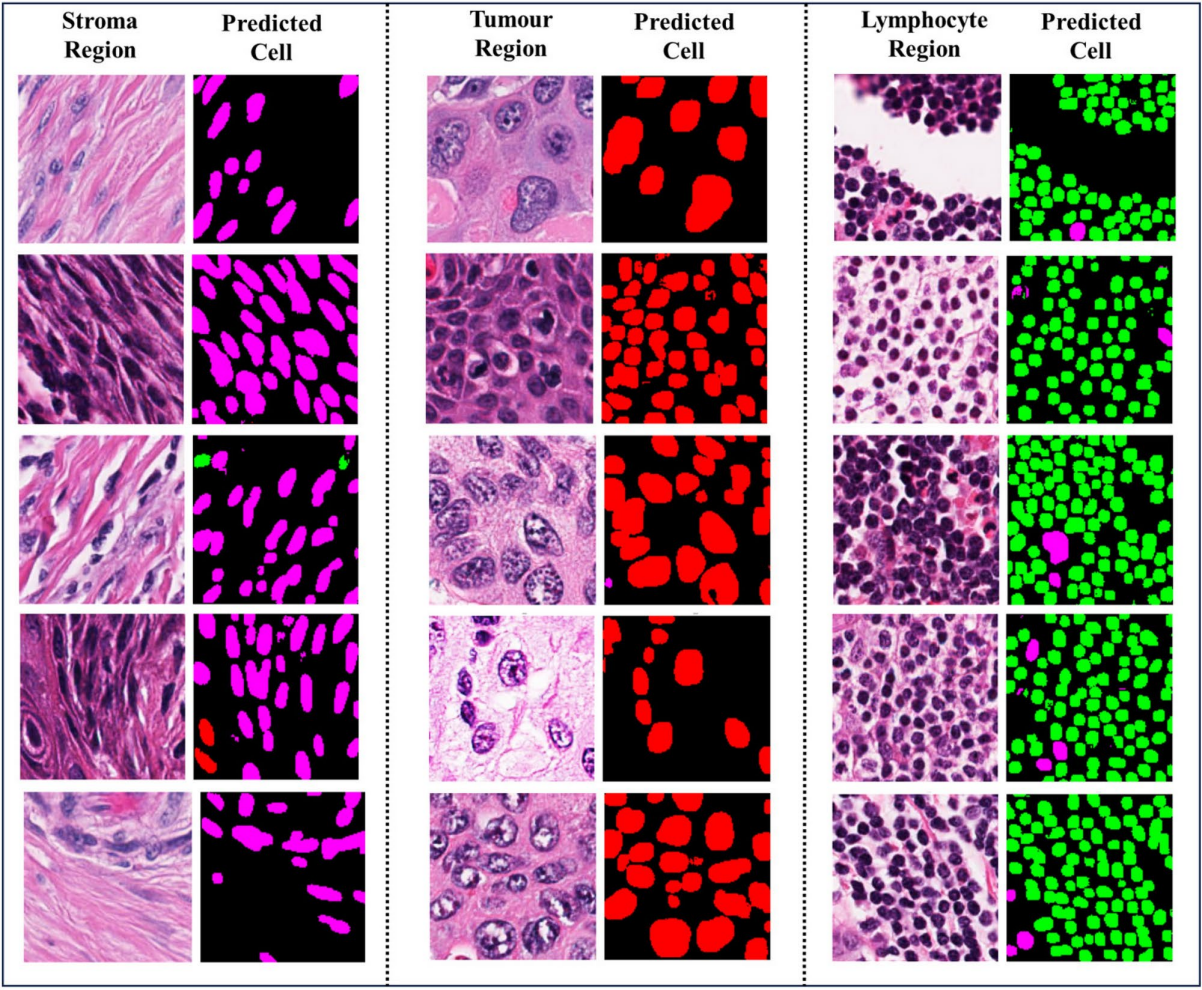
Generalization test visual interpretation (Red color represents Tumour cells, Green color represents Lymphocyte/plasma cell and Dark pink color represents Stroma cells).

To conduct this test, we selected tissue patches from the *OSCC*$_{tcga}$ dataset that represent tumour, stroma, and lymphocyte-rich regions. After segmenting these patches, we calculated the pixel density of each cell type within them. Based on the highest cell type density, we classified each patch. For example, a patch with the highest density of stroma cells is labelled as “stroma region”. Similarly, a patch with the highest density of tumour cells and lymphocyte cell/plasma cells is labelled as Tumour and Lymphocyte region (TILs representative) respectively. We then compared these predicted labels with the original labels provided by pathologists. This allowed us to assess the proposed model’s performance using metrics: Accuracy, precision, recall and F1 score and we have also performed a visual inspection of the generated result as shown in the Fig. 5.

The Table 3 provides the details of the generalization test of proposed segmentation model for each cellular composition

**Table 3:** Generalization test analysis of proposed model on *OSCC*$_{tcga}$.

Cell type	Accuracy	Precision	Recall	F1-score
Stroma	96.55	93.96	96.55	95.24
TILs	92.14	97.73	92.14	94.85
Tumour	98.86	96.65	98.86	97.74

The results in Table 3 demonstrate that the proposed segmentation model generalizes effectively on the *OSCC*$_{tcga}$ dataset without fine-tuning. The model achieves high accuracy, precision, recall, and F1 scores across all cell types, highlighting its robustness.

In tumour regions, the model shows outstanding performance with an accuracy of 0.989, precision of 0.966, recall of 0.989, and an F1-score of 0.977. This indicates that the model is highly effective at correctly identifying tumour cells with minimal false negatives.

In stroma regions, the model achieves a high accuracy of 0.966, with a precision of 0.940 and a recall of 0.966, resulting in an F1-score of 0.952. This balance between precision and recall suggests the model accurately detects stromal cells while minimizing both false positives and false negatives.

For lymphocyte-rich regions, the model performs well with an accuracy of 0.921, a precision of 0.977, and a recall of 0.921, leading to an F1-score of 0.949. The high precision here indicates that the model effectively identifies lymphocyte/TILs cells.

Overall, the proposed model’s average precision, recall, and F1-scores of 0.961, 0.959, and 0.959, respectively, indicate its consistent ability to generalize across all cell types in the *OSCC*$_{tcga}$ dataset.

Figure 5 shows that our proposed model accurately predicts cellular distributions specific to different tissue regions. For example, in the Stroma region patch, where Stroma cells are most dense, the proposed method generates a segmentation mask reflecting this high density of Stroma cells. Similarly, in the Tumour and Lymphocyte regions, the model predicts cellular distributions that closely align with the characteristics of each specific tissue region.

These results confirm the model’s accuracy, reliability, and strong generalization capability. This suggests that the proposed model can generate accurate pseudo masks of *OSCC*$_{tcga}$ dataset, which can then be used to fine-tune the model further for even more precise segmentation.

### Self consistency test of proposed segmentation model

To evaluate the segmentation accuracy and capability of our model after fine-tuning on the pseudo mask of *OSCC*$_{tcga}$ dataset, we performed a self-consistency test. This test is essential for assessing the model’s performance and stability without relying on detailed ground truth masks. It provides valuable insights into the model’s ability to handle variations and generalize effectively. The self-consistency test involves augmenting the *OSCC*$_{tcga}$ test set using two different augmentation methods. The first augmentation applies horizontal rotations, and the second adds Gaussian noise. These augmentations create two distinct versions of the test set. The model then generates segmentation masks for both the augmented and non-augmented test sets. We compare the generated segmentation masks between augmented instances and non-augmented test set instances to evaluate the model’s consistency. High consistency between the generated masks indicates that the model is robust and capable of generalizing well across different variations of the data. To quantify this self-consistency, we use the Structural Similarity Index (SSIM)^32^ metric to measure the similarity of structural features and Intersection over Union (IoU) to assess the overlap between the generated instances. The SSIM measures the similarity between two images by comparing their structural information, luminance, and contrast. SSIM can be calculated as shown in the Eq. (5)

5 $$\begin{aligned} \text {SSIM}(x, y) = \frac{(2\mu _x \mu _y + C_1)(2\sigma _{xy} + C_2)}{(\mu _x^2 + \mu _y^2 + C_1)(\sigma _x^2 + \sigma _y^2 + C_2)} \end{aligned}$$

where $\mu _x$ and $\mu _y$ represent the mean pixel intensities of the non augmented and augmented generated segmentation masks, respectively. $\sigma _x^2$ and $\sigma _y^2$ denote the variances of the non augmented and augmented generated masks. $\sigma _{xy}$ is the covariance between the non augmented and augmented masks. $C_1$ and $C_2$ are constants that stabilize the division when the denominator is small.

**Table 4:** Self consistency analysis of test set of *OSCC*$_{tcga}$ dataset (Average value).

Consistency test	IoU	SSIM
Gaussian augmented vs Test set	0.963	0.997
Horizontal augmented vs Test set	0.721	0.963

**Fig. 6:**
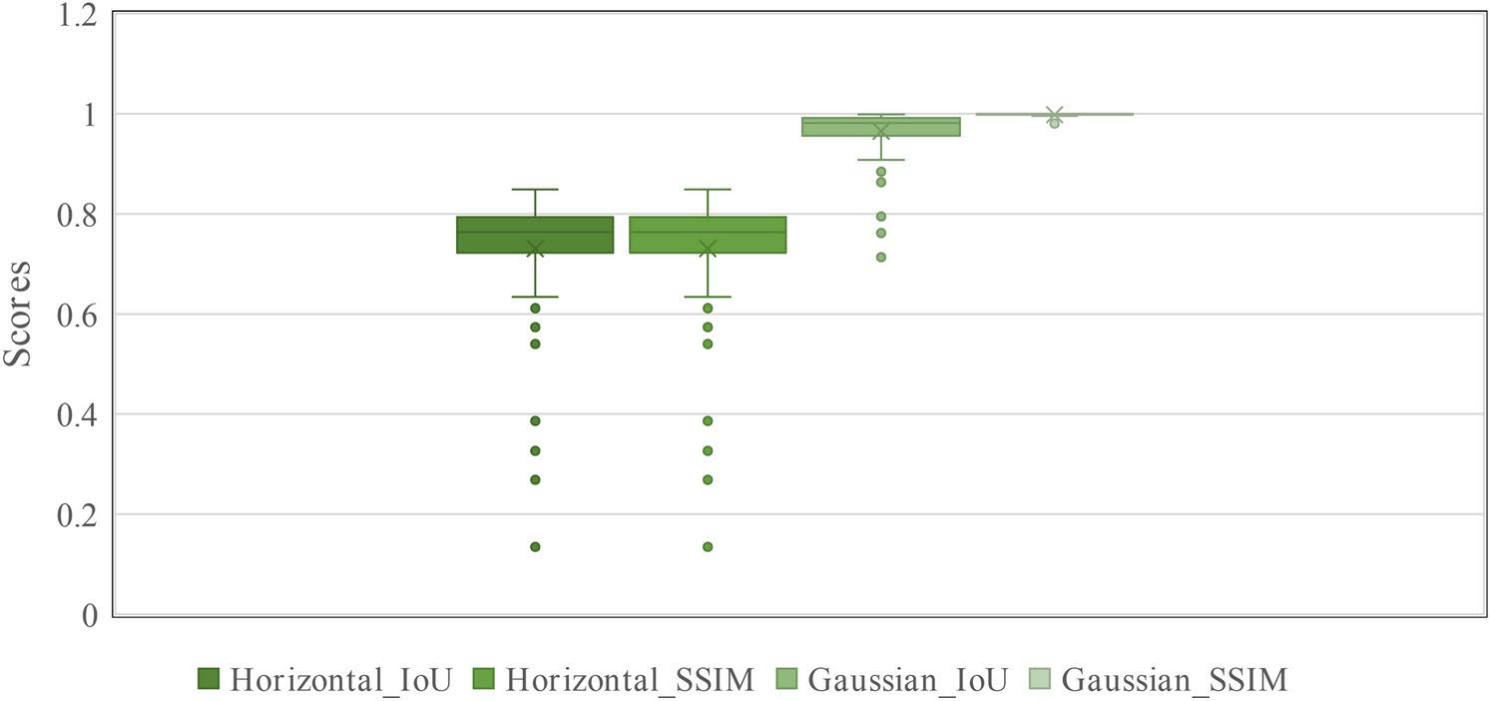
Consistency test IOU and SSIM score’s box plot over 100 images of test set of *OSCC*$_{tcga}$. Here *Horizontal_IoU* and *Horizontal_SSIM* represent the score obtained after the comparison of Horizontal augmented and non-augmented test set instances. And *Gaussian_IoU* and *Gaussian_SSIM* represent the score obtained after the comparison of Gaussian augmented and non-augmented test set instances.

Table 4 shows the results of the self-consistency analysis for the *OSCC*$_{tcga}$ dataset, using IoU and SSIM metrics. For the Gaussian noise-augmented vs non-augmented test set, the model achieved a high IoU of 0.963 and an SSIM of 0.997, demonstrating strong performance and robustness in handling noise while maintaining excellent segmentation accuracy and structural similarity. For the horizontal rotation-augmented and non-augmented test set, the model recorded an IoU of 0.721 and an SSIM of 0.963. Although the IoU is slightly lower, it still indicates a good level of overlap between the two instances. The high SSIM confirms that the model maintains strong structural consistency despite the rotational variations.

Figure 6 presents the box plot of 100 images for a better understanding of the distribution of scores of self consistency test.

From the result we can say overall the model performs effectively with both types of augmentation, achieving high IoU and SSIM values. These results indicate that the proposed model is robust and exhibits strong segmentation performance.

**Fig. 7:**
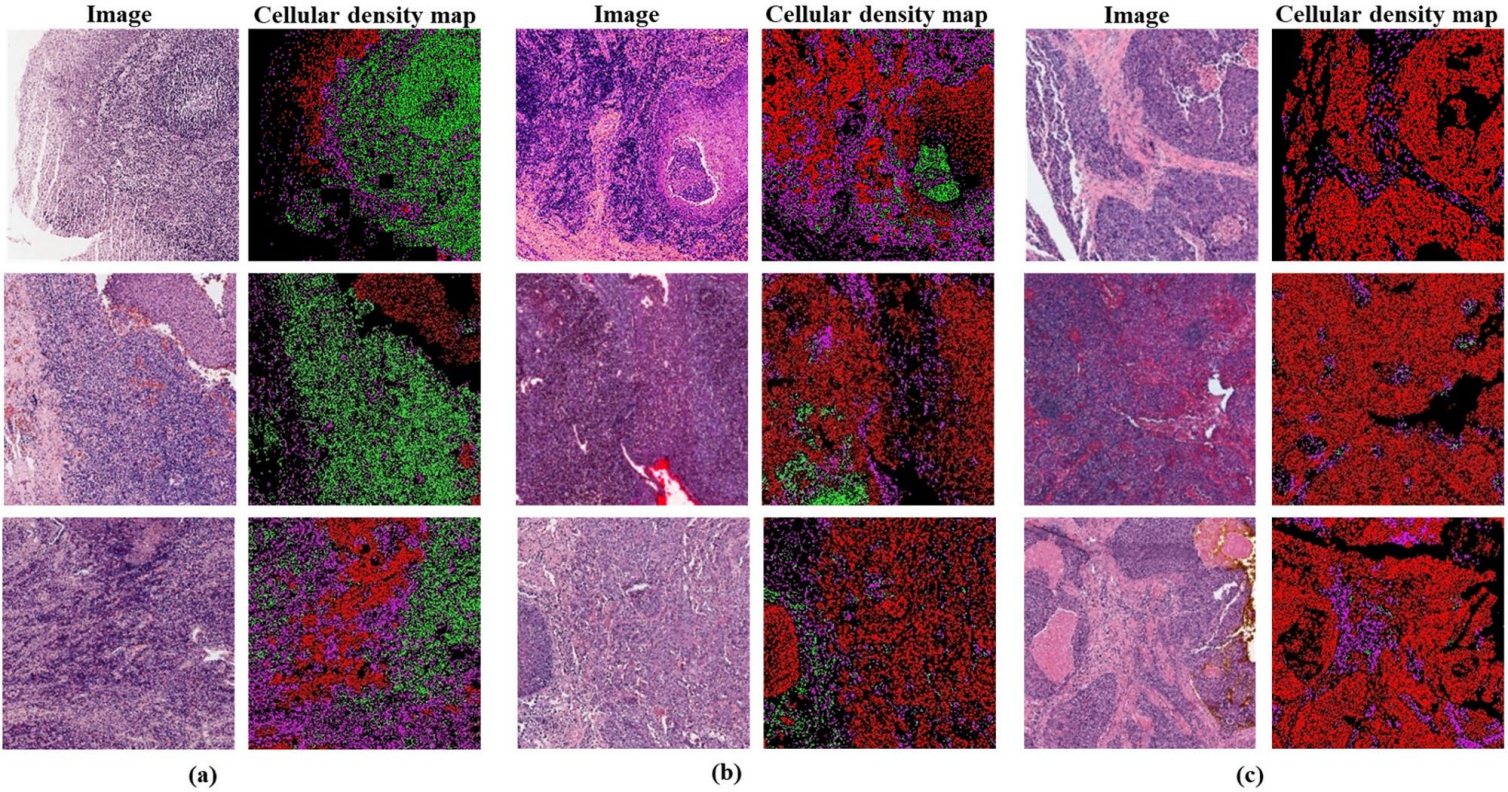
Examples of cellular density map of large FOV patches of each class of TILs: (**a**) Moderate to Marked, (**b**) Slight, (**c**) None to very less (Red color represents Tumour cells, Green color represents Lymphocyte/plasma cell, Dark pink color represents Stroma cells).

Based on the tests conducted above, we have found that the proposed segmentation model, TILSeg-MobileViT, is capable of generating accurate segmentation masks. Therefore, it can be used to produce large FOV patches cellular density maps, which will serve as input for our proposed joint representation TILs classifier, OralTILs-ViT. Figure 7 illustrates some examples of generated cellular density maps of large FOV patches arranged according to TILs presence.

In the following section, we evaluate the performance of OralTILs-ViT in terms of TILs classification.

### TILs classification results

This section presents the result of the performance evaluation of our proposed joint representation classifier OralTILs-ViT. For evaluation, the proposed classifier is compared with single representation learners i.e, $\phi _{img}$ (only image) and $\phi _{cell}$ (only cell density map), where $\phi _{img}$ (only image) can be the representative of patch-based techniques^13,14^. This comparison highlights the advantages of our joint representation approach over individual representation learning methods. The evaluation metrics used include accuracy, precision, recall, and F1-score, providing a comprehensive assessment of model performance. Additionally, we investigated the impact of hyperparameter configurations on the combination of learning rates($\alpha$ = 0.001 and 0.0001) and optimizers (SGD and Adam), on performance. An error analysis was conducted for each TILs class for each hyperparameter configuration by examining confusion matrices, enabling insights into the model’s classification errors. Furthermore, to identify the best-performing method with hyperparameter configuration based on the performance metrics, we applied a multi-criteria decision analysis technique, specifically the Technique for Order of Preference by Similarity to Ideal Solution (TOPSIS), using the Entropy Weighting methodology.

**Table 5:** Performance comparison of methods across TILs classes under different hyperparameter settings. The hyperparameter used are Learning rates ($\alpha$): 0.001 *and *0.0001 with two different *Optimizers*: *SGD and Adam*. Significant values are in bold.

Classes	Methods	Accuracy	Precision	Recall	F1-score
None to Very less TILs	Only Image ($\alpha =0.001, Optimizer= Adam$)	93.85%	79.09%	93.85%	85.84%
	Only Image ($\alpha =0.0001, Optimizer=Adam$)	83.85%	82.73%	83.85%	83.29%
	Only Image ($\alpha =0.001, Optimizer=SGD$)	86.35%	86.35%	86.35%	86.35%
	Only Image ($\alpha =0.0001, Optimizer=SGD$)	86.92%	83.24%	86.92%	85.04%
	Only Cell density map ($\alpha =0.001, Optimizer=Adam$)	95.77%	97.08%	95.77%	96.42%
	Only Cell density map ($\alpha =0.0001, Optimizer=Adam$)	98.08%	92.06%	98.08%	94.97%
	Only Cell density map ($\alpha =0.001, Optimizer=SGD$)	96.35%	94.00%	96.35%	95.16%
	Only Cell density map ($\alpha =0.0001, Optimizer=SGD$)	97.50%	95.30%	97.50%	96.39%
	OralTILs-ViT ($\alpha =0.001, Optimizer=Adam$)	96.73%	97.10%	96.73%	96.92%
	OralTILs-ViT ($\alpha =0.0001, Optimizer=Adam$)	97.69%	91.70%	97.69%	94.60%
	OralTILs-ViT ($\alpha =0.001, Optimizer=SGD$)	96.35%	95.98%	96.35%	96.16%
	OralTILs-ViT ($\alpha =0.0001, Optimizer=SGD$)	97.88%	94.09%	97.88%	95.95%
Slight TILs	Only Image ($\alpha =0.001, Optimizer=Adam$)	69.57%	80.13%	69.57%	74.48%
	Only Image ($\alpha =0.0001, Optimizer=Adam$)	70.93%	69.85%	70.93%	70.38%
	Only Image ($\alpha =0.001, Optimizer=SGD$))	64.53%	76.38%	64.53%	69.96%
	Only Image ($\alpha =0.0001, Optimizer=SGD$)	70.35%	70.21%	70.35%	70.28%
	Only Cell density map ($\alpha =0.001, Optimizer=Adam$)	95.35%	90.61%	95.35%	92.92%
	Only Cell density map ($\alpha =0.0001, Optimizer=Adam$)	88.37%	92.49%	88.37%	90.39%
	Only Cell density map ($\alpha =0.001, Optimizer=SGD$)	92.83%	89.37%	92.83%	91.06%
	Only Cell density map ($\alpha =0.0001, Optimizer=SGD$)	89.34%	95.05%	89.34%	92.11%
	OralTILs-ViT ($\alpha =0.001, Optimizer=Adam$)	94.57%	94.76%	94.57%	94.67%
	OralTILs-ViT ($\alpha =0.0001, Optimizer=Adam$)	88.57%	93.65%	88.57%	91.04%
	OralTILs-ViT ($\alpha =0.001, Optimizer=SGD$)	95.16%	89.44%	95.16%	92.21%
	OralTILs-ViT ($\alpha =0.0001, Optimizer=SGD$)	91.47%	93.28%	91.47%	92.37%
Moderate to Marked TILs	Only Image ($\alpha =0.001, Optimizer=Adam$)	85.90%	91.76%	85.90%	88.74%
	Only Image ($\alpha =0.0001, Optimizer=Adam$)	82.60%	85.42%	82.60%	83.99%
	Only Image ($\alpha =0.001, Optimizer=SGD$))	92.51%	78.65%	92.51%	85.02%
	Only Image ($\alpha =0.0001, Optimizer=SGD$)	80.40%	84.88%	80.40%	82.58%
	Only Cell density map ($\alpha =0.001, Optimizer=Adam$)	93.61%	97.93%	93.61%	95.72%
	Only Cell density map ($\alpha =0.0001, Optimizer=Adam$)	94.05%	96.39%	94.05%	95.21%
	Only Cell density map ($\alpha =0.001, Optimizer=SGD$)	91.63%	98.81%	91.63%	95.09%
	Only Cell density map ($\alpha =0.0001, Optimizer=SGD$)	97.58%	93.66%	97.58%	95.58%
	OralTILs-ViT ($\alpha =0.001, Optimizer=Adam$)	97.80%	97.16%	97.80%	97.48%
	OralTILs-ViT ($\alpha =0.0001, Optimizer=Adam$)	95.81%	97.10%	95.81%	96.45%
	OralTILs-ViT ($\alpha =0.001, Optimizer=SGD$)	91.41%	99.05%	91.41%	95.07%
	OralTILs-ViT ($\alpha =0.0001, Optimizer=SGD$)	94.93%	97.29%	94.93%	96.10%
Overall	Only Image ($\alpha =0.001, Optimizer=Adam$)	83.11%	83.66%	83.11%	83.02%
	Only Image ($\alpha =0.0001, Optimizer=Adam$)	79.13%	79.33%	79.13%	79.22%
	Only Image ($\alpha =0.001, Optimizer=SGD$)	81.13%	80.46%	81.13%	80.44%
	Only Image ($\alpha =0.0001, Optimizer=SGD$)	79.22%	79.45%	79.22%	79.30%
	Only Cell density map ($\alpha =0.001, Optimizer=Adam$)	94.91%	95.20%	94.91%	95.02%
	Only Cell density map ($\alpha =0.0001, Optimizer=Adam$)	93.50%	93.65%	93.50%	93.52%
	Only Cell density map ($\alpha =0.001, Optimizer=SGD$)	93.60%	94.06%	93.60%	93.77%
	Only Cell density map ($\alpha =0.0001, Optimizer=SGD$)	94.81%	94.67%	94.81%	94.69%
	**OralTILs-ViT ** ($\alpha =0.001, Optimizer=Adam$)	**96.37**%	**96.34**%	**96.37**%	**96.35**%
	OralTILs-ViT ($\alpha =0.0001, Optimizer=Adam$)	94.02%	94.15%	94.02%	94.03%
	OralTILs-ViT ($\alpha =0.001, Optimizer=SGD$)	94.30%	94.82%	94.30%	94.48%
	OralTILs-ViT ($\alpha =0.0001, Optimizer=SGD$)	94.76%	94.89%	94.76%	94.80%

#### Performance comparison analysis

Table 5 summarizes the comparison of three methods for TILs classification: using only image features ($\phi _{img}$), only cell density maps ($\phi _{cell}$), and the proposed OralTILs-ViT model, which employs a joint representation of both features. The evaluation metrics-accuracy, precision, recall, and F1-score-are reported across different TILs categories: None to Very Less, Slight, Moderate to Marked, and Overall. Additionally, the table highlights the impact of various hyperparameter configurations, including learning rates and optimizers, on the performance of each method.

From Table 5 we can observed the OralTILs-ViT, which integrates both raw image features and cellular density map features, consistently outperformed both individual models (Only Cell Density Map ($\phi _{\text {cell}}$) and Only Image ($\phi _{\text {img}}$)) across all TILs categories and metrics. This fusion approach proved highly effective in improving overall model performance. The highest performance metrics for OralTILs-ViT were achieved with Adam optimizer ($\alpha$ = 0.001), with **96.37% accuracy**, **96.34% precision**, **96.37% recall**, and an **F1-score of 96.35%**.

For the None to Very Less TILs category, the OralTILs-ViT model significantly outperformed both the Only Image ($\phi _{\text {img}}$) and Only Cell Density Map ($\phi _{\text {cell}}$) models. The best performance for OralTILs-ViT was observed with Adam ($\alpha$ = 0.001), yielding an accuracy of 96.73%, precision of 97.10%, recall of 96.73%, and an F1-score of 96.92%. In comparison, the Only Cell Density Map ($\phi _{\text {cell}}$) model achieved an accuracy of 98.08%, precision of 92.06%, recall of 98.08%, and an F1-score of 94.97% using Adam ($\alpha$ = 0.0001). Meanwhile, the Only Image ($\phi _{\text {img}}$) model recorded a significantly lower F1-score of 85.84%, with an accuracy of 93.85%, precision of 79.09%, and recall of 93.85% using Adam ($\alpha$ = 0.001).

In the *Slight* TILs category, the OralTILs-ViT model again outperformed both individual models, with the highest performance metrics achieved using Adam ($\alpha$ = 0.001), resulting in 94.57% accuracy, 94.76% precision, 94.57% recall, and an F1-score of 94.67%. The Only Cell Density Map ($\phi _{\text {cell}}$) model, with Adam ($\alpha$ = 0.001), achieved 95.35% accuracy, 90.61% precision, 95.35% recall, and an F1-score of 92.92%, while the Only Image ($\phi _{\text {img}}$) model demonstrated much lower performance, with an accuracy of 70.93%, precision of 80.13%, recall of 69.57%, and an F1-score of 74.48% using Adam ($\alpha$ = 0.001).

For the *Moderate to Marked* TILs category, the OralTILs-ViT model once again delivered the highest performance. Using Adam ($\alpha$ = 0.001), it achieved 97.80% accuracy, 97.16% precision, 97.80% recall, and an F1-score of 97.48%. While the Only Cell Density Map ($\phi _{\text {cell}}$) model performed well, with an F1-score of 95.72%,accuracy of 93.61%, precision of 97.93%, recall of 93.61% using Adam ($\alpha$ = 0.001), the Only Image ($\phi _{\text {img}}$) model was comparatively weaker, with an accuracy of 92.51%, precision of 78.65%, recall of 92.51%, and an F1-score of 85.02%, achieved using SGD ($\alpha$ = 0.001).

This comprehensive performance evaluation establishes that the OralTILs-ViT model outperforms other other model. And from this evaluation we can establish that the best-performing hyperparameter configuration across all categories and models is Adam optimizer with a learning rate ($\alpha$) of 0.001. This configuration consistently delivered strong performance across the various models, including OralTILs-ViT, Only Image, and Only Cell Density Map, yielding high accuracy, precision, recall, and F1-scores.

#### Multi-criteria decision analysis (MCDA) for obtaining best performing model

To further validated and identify the best-performing model with best hyperparameter configuration across all categories of TILs, we applied a multi-criteria decision analysis (MCDA) for assessment^33–35^. Specifically, we utilized the Technique for Order of Preference by Similarity to Ideal Solution (TOPSIS), in conjunction with the Information Entropy Weighting Methodology^36^. This approach enabled us to assess the models using multiple criteria, including accuracy, precision, recall, and F1-score, particularly given the similarity of certain performance metrics across the models.

Table 6 provides a detailed evaluation of three TILs classification methods with different hyperparameter configuration—OralTILs-ViT (Joint representation), Only Image ($\phi _{\text {img}}$) and Only Cell Density Map ($\phi _{\text {cell}}$)-using TOPSIS scores and rankings across TILs categories: None to Very Less, Slight, and Moderate to Marked. The analysis clearly shows the consistent superiority of the proposed OralTILs-ViT (Joint representation) method.

In the None to Very Less TILs category, OralTILs-ViT (Adam, $\alpha$ = 0.001) ranked first with a TOPSIS score of 0.952, significantly outperforming the second-best model, Only Cell Density Map (SGD, $\alpha$ = 0.0001), which scored 0.920. The Only Image models performed poorly, with TOPSIS scores well below 0.4.

In the Slight TILs category, OralTILs-ViT (Adam, $\alpha$ = 0.001) again showed outstanding performance, achieving the highest score of 0.978, followed by Only Cell Density Map (Adam, $\alpha$ = 0.001) with a score of 0.941. The Only Image models continued to rank lowest, with scores not exceeding 0.2, highlighting their limited ability to classify this category effectively.

For the Moderate to Marked TILs category, OralTILs-ViT (Adam, $\alpha$ = 0.001) retained its top rank with a score of 0.937. However, the competition was closer in this category. OralTILs-ViT (Adam, $\alpha$ = 0.0001) secured second place with a score of 0.899, while OralTILs-ViT (SGD, $\alpha$ = 0.0001) ranked third with a score of 0.877. The Only Cell Density Map models showed moderate performance, with scores between 0.786 and 0.842.

Overall, **OralTILs-ViT (Adam,** $\alpha = 0.001$) **achieved the highest TOPSIS score of 1** across all categories, making it the most effective and consistent configuration for TILs classification. The Only Cell Density Map (Adam, $\alpha$ = 0.001) model ranked second overall with a score of 0.921, demonstrating the utility of cellular density maps. In contrast, the Only Image models consistently ranked last across all categories, with scores below 0.3, indicating their limited standalone classification capability.

These results further validate the robustness of our proposed joint representation learning model, OralTILs-ViT, which significantly outperformed single-modality models such as Only Image ($\phi _{\text {img}}$) and Only Cell Density Map ($\phi _{\text {cell}}$) in classifying TILs in larger field-of-view histopathology images.

**Table 6:** Multi-criteria decision analysis based on TOPSIS to obtain best performing method with best hyperparameter configuration across all classes of TILs. Significant values are in bold.

Classes	Methods	TOPSIS_Score	Rank
None to Very less TILs	**OralTILs-ViT** ($\alpha =0.001, Optimizer=Adam$)	**0.952**	**1**
	Only Cell density map ($\alpha =0.0001, Optimizer=SGD$)	0.920	2
	OralTILs-ViT ($\alpha =0.001, Optimizer=SGD$)	0.919	3
	Only Cell density map ($\alpha =0.001, Optimizer=Adam$)	0.918	4
	OralTILs-ViT ($\alpha =0.0001, Optimizer=SGD$)	0.873	5
	Only Cell density map ($\alpha =0.001, Optimizer=SGD$)	0.847	6
	Only Cell density map ($\alpha =0.0001, Optimizer=Adam$)	0.790	7
	OralTILs-ViT ($\alpha =0.0001, Optimizer=Adam$)	0.772	8
	Only Image ($\alpha =0.001, Optimizer=SGD$)	0.323	9
	Only Image ($\alpha =0.001, Optimizer=Adam$)	0.308	10
	Only Image ($\alpha =0.0001, Optimizer=SGD$)	0.212	11
	Only Image ($\alpha =0.0001, Optimizer=Adam$)	0.147	12
Slight TILs	**OralTILs-ViT** ($\alpha =0.001, Optimizer=Adam$)	**0.978**	**1**
	Only Cell density map ($\alpha =0.001, Optimizer=Adam$)	0.941	2
	OralTILs-ViT ($\alpha =0.001, Optimizer=SGD$)	0.924	3
	Only Cell density map ($\alpha =0.001, Optimizer=SGD$)	0.887	4
	OralTILs-ViT ($\alpha =0.0001, Optimizer=SGD$)	0.883	5
	Only Cell density map ($\alpha =0.0001, Optimizer=SGD$)	0.828	6
	OralTILs-ViT ($\alpha =0.0001, Optimizer=Adam$)	0.801	7
	Only Cell density map ($\alpha =0.0001, Optimizer=Adam$)	0.790	8
	Only Image ($\alpha =0.001, Optimizer=Adam$)	0.200	9
	Only Image ($\alpha =0.0001, Optimizer=Adam$)	0.177	10
	Only Image ($\alpha =0.0001, Optimizer=SGD$)	0.162	11
	Only Image ($\alpha =0.001, Optimizer=SGD$)	0.076	12
Moderate to Marked TILs	**OralTILs-ViT** ($\alpha =0.001, Optimizer=Adam$)	**0.937**	**1**
	OralTILs-ViT ($\alpha =0.0001, Optimizer=Adam$)	0.899	2
	OralTILs-ViT ($\alpha =0.0001, Optimizer=SGD$)	0.877	3
	Only Cell density map ($\alpha =0.001, Optimizer=Adam$)	0.843	4
	Only Cell density map ($\alpha =0.0001, Optimizer=Adam$)	0.829	5
	Only Cell density map ($\alpha =0.0001, Optimizer=SGD$)	0.820	6
	Only Cell density map ($\alpha =0.001, Optimizer=SGD$)	0.786	7
	OralTILs-ViT ($\alpha =0.001, Optimizer=SGD$)	0.781	8
	Only Image ($\alpha =0.001, Optimizer=Adam$)	0.486	9
	Only Image ($\alpha =0.001, Optimizer=SGD$)	0.363	10
	Only Image ($\alpha =0.0001, Optimizer=Adam$)	0.238	11
	Only Image ($\alpha =0.0001, Optimizer=SGD$)	0.196	12
Overall	**OralTILs-ViT**($\alpha =0.001, Optimizer=Adam$)	**1.000**	**1**
	Only Cell density map ($\alpha =0.001, Optimizer=Adam$)	0.921	2
	OralTILs-ViT ($\alpha =0.0001, Optimizer=SGD$)	0.909	3
	Only Cell density map ($\alpha =0.0001, Optimizer=SGD$)	0.906	4
	OralTILs-ViT ($\alpha =0.001, Optimizer=SGD$)	0.890	5
	OralTILs-ViT ($\alpha =0.0001, Optimizer=Adam$)	0.866	6
	Only Cell density map ($\alpha =0.001, Optimizer=SGD$)	0.848	7
	Only Cell density map ($\alpha =0.0001, Optimizer=Adam$)	0.836	8
	Only Image ($\alpha =0.001, Optimizer=Adam$)	0.235	9
	Only Image ($\alpha =0.001, Optimizer=SGD$)	0.095	10
	Only Image ($\alpha =0.0001, Optimizer=SGD$)	0.006	11
	Only Image ($\alpha =0.0001, Optimizer=Adam$)	0.000	12

#### Error analysis and interpretation

**Fig. 8:**
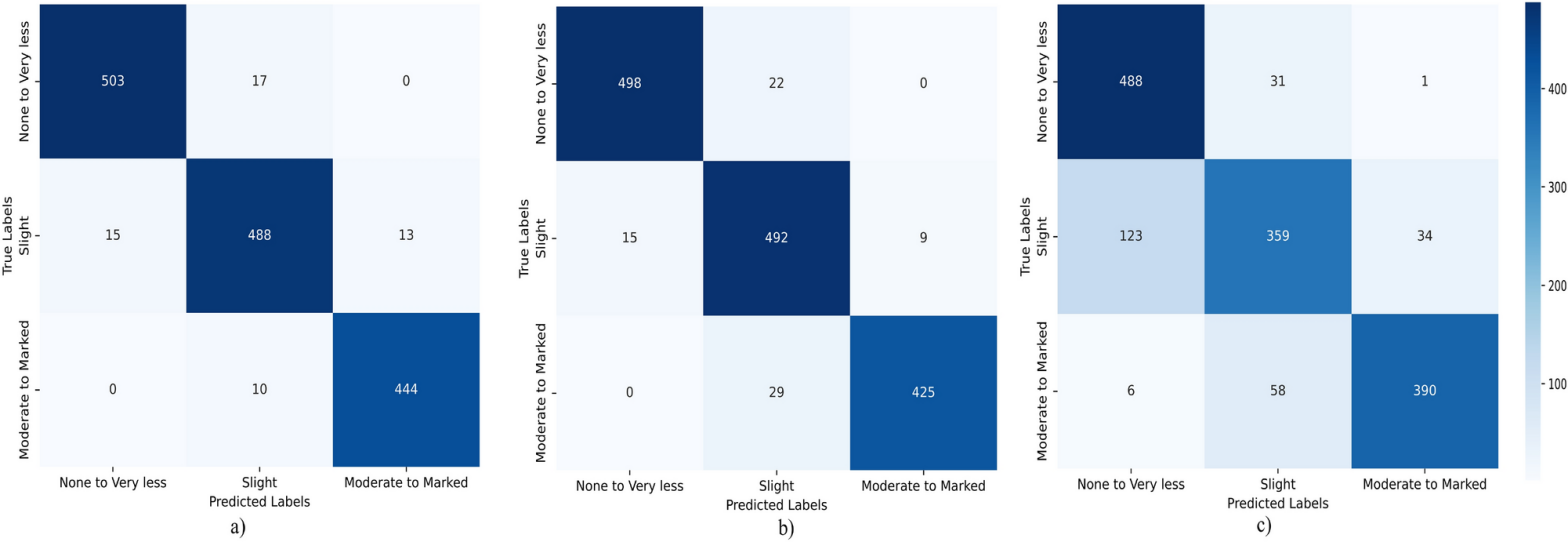
Confusion matrices of (**a**) Oral-vit (Joint representation), (**b**) Only Cell density map ($\phi _{cell}$), (**c**) Only Image ($\phi _{img}$).

An error analysis was conducted on the best-performing models for each modality-Only Image ($\phi _{\text {img}}$) with the configuration (Adam, $\alpha$ = 0.001), Only Cell Density Map ($\phi _{\text {cell}}$) with the configuration (Adam, $\alpha$ = 0.001), and Joint Representation (OralTILs-ViT) with the configuration (Adam, $\alpha$ = 0.001)-to further evaluate their classification performance across the three TILs categories: None to Very Less, Slight, and Moderate to Marked. This analysis, summarized in Fig. 8a–c, provides valuable insights into the trends and misclassification patterns of these models, highlighting the advantages of the joint representation approach.

From Fig. 8a–c, the following trends were observed across the TILs categories:

- None to Very Less: The Only Image ($\phi _{\text {img}}$) model exhibited the highest misclassification rate, with a significant number of false positives. The proposed OralTILs-ViT model achieved the best performance in this category, with 503 true positives (TP), compared to 498 TP for the Only Cell Density Map ($\phi _{\text {cell}}$) model and 488 TP for the Only Image ($\phi _{\text {img}}$) model.
- Slight: The Only Image ($\phi _{\text {img}}$) model again showed the highest misclassification rate in this category. In contrast, the Only Cell Density Map ($\phi _{\text {cell}}$) model and the OralTILs-ViT model demonstrated comparable performance, achieving 492 TP and 488 TP, respectively.
- Moderate to Marked: The OralTILs-ViT model exhibited the lowest misclassification rate in this category, with 444 TP, significantly outperforming the Only Image ($\phi _{\text {img}}$) model (390 TP) and the Only Cell Density Map ($\phi _{\text {cell}}$) model (425 TP). This highlights the superior accuracy of the joint representation approach in this category.

Overall, the proposed OralTILs-ViT model consistently outperformed the single-modality models, Only Image ($\phi _{\text {img}}$) and Only Cell Density Map ($\phi _{\text {cell}}$), across all TILs categories. The joint representation approach minimized misclassification and demonstrated robust and accurate performance, making it the most effective method for TILs classification.

**Fig. 9:**
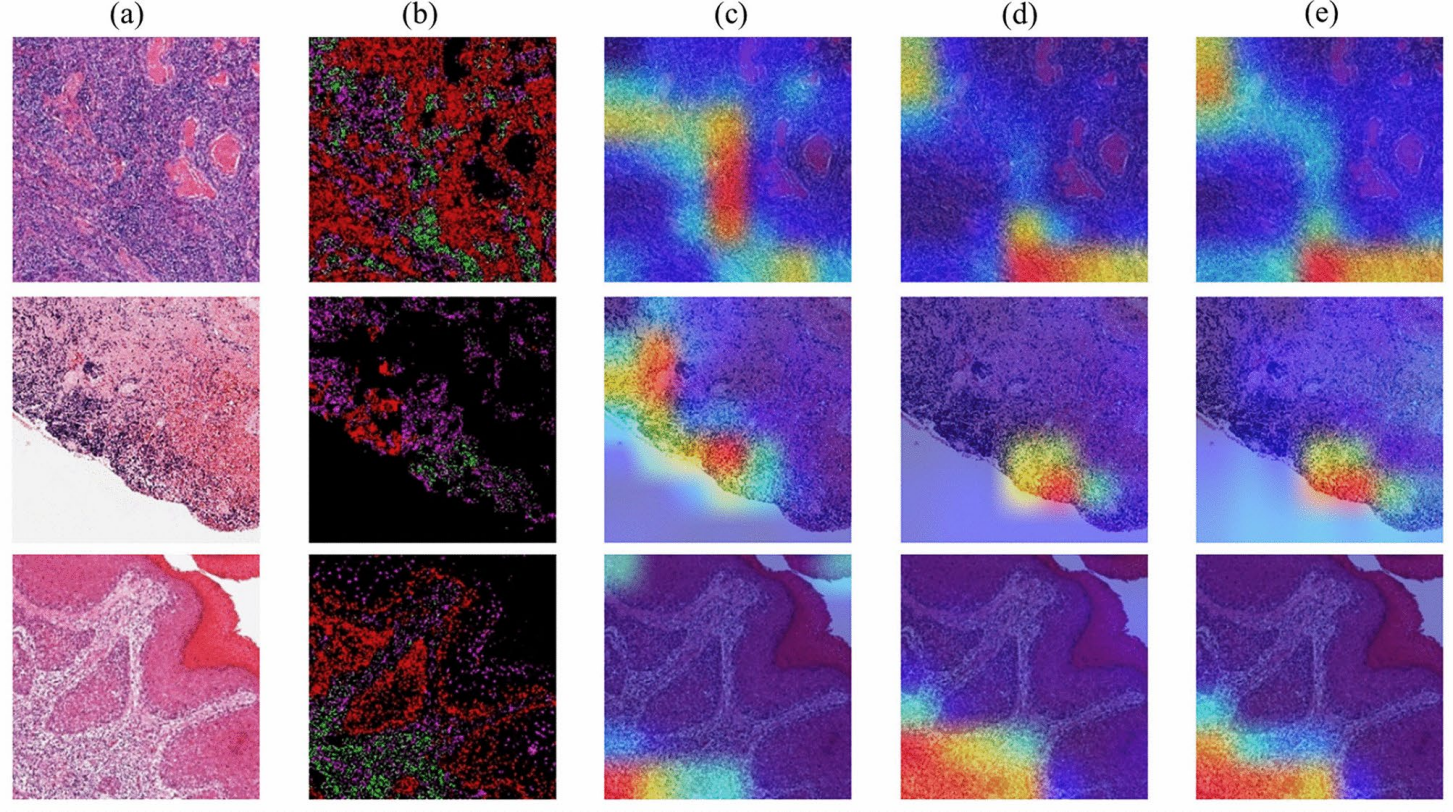
Grad-CAMs interpretation of methods. (**a**) Image, (**b**) Cellular density map, Grad-CAMs generated by: (**c**) $\phi _{img}$, (**d**) $\phi _{cell}$, (**e**) OralTILs-ViT.

Additionally, Fig. 9 illustrates presents some examples of Grad-CAMs^37^ interpretation of correctly classified test set samples from the three methods-OralTILs-ViT (Joint representation), Only Cell density map ($\phi _{cell}$), and Only Image ($\phi _{img}$). It can be observed that OralTILs-ViT consistently focuses on the correct TILs regions for classification, demonstrating its effectiveness in accurately identifying relevant areas.

## Discussion and conclusion

In this study, we introduced OralTILs-ViT, a novel framework for the multiclass classification of TILs in histopathology images of OSCC, addressing the need for more accurate and objective TILs quantification. TILs, a key pathological feature, reflect the immune system’s response to cancer and hold significant prognostic value in OSCC. However, current methods for TILs assessment, such as those by Shaban *et al.*, rely on patch-level analysis that focuses on tissue-level spatial information while neglecting the critical cellular semantics required for accurate TIL quantification. This limitation hampers the capture of complex cellular interactions and spatial arrangements essential for thorough TIL analysis.

Our framework addresses these challenges by integrating both cellular and tissue-level information. Specifically, OralTILs-ViT leverages a weakly supervised cellular density map, combined with H&E-stained tissue images, to classify the peritumoral stroma into categories aligned with Broders’ grading system-“Moderate to Marked”, “Slight” and “None to Very Less”. By capturing both tissue and cellular relationships, especially in the peritumoral stroma, this approach better mirrors clinical pathology practices, offering a significant improvement in TILs classification. The methodology consists of two stages. In the first stage, we generate a cellular density map of OSCC tissue patches using our proposed multiclass semantic segmentation model, TILSeg-MobileViT, which focuses on key cellular components such as tumour cells, stromal cells, and lymphocytes. In the second stage, we classify the peritumoral stroma based on TILs presence, utilizing a joint representation TIL classifier, OralTILs-ViT.

To the best of our knowledge, this is the first study to perform multiclass subtyping of TILs presence in OSCC using a joint representation of cellular density maps and H&E-stained images. This approach contrasts with existing methods that rely solely on patch-level tissue information and fail to capture critical cellular details, offering a more comprehensive and accurate TILs analysis.

In this study, we conducted a comprehensive evaluation of our proposed methodology, which can be divided into multiple stages. First, we compared our segmentation model, TILSeg-MobileViT, with UNet and Attention-UNet using IoU and Dice metrics. As shown in the results, our model outperformed the others in segmentation. We also assessed its generalization capability using accuracy, precision, recall, and F1 score, with high values confirming its robustness, supported by visual inspection. After fine-tuning the OSCC dataset, we performed a self-consistency test to validate performance without ground-truth masks, demonstrating high segmentation accuracy and generalization. Next, we evaluated our TILs classifier, OralTILs-ViT, using accuracy, precision, recall, and F1 score, comparing it to single-modality models—Only Image ($\phi _{\text {img}}$) and Only Cell Density Map ($\phi _{\text {cell}}$), along with hyperparameter impact analysis. This comparison highlights the superiority of our joint representation approach. OralTILs-ViT with the configuration (Adam, $\alpha$ = 0.001) achieved **96.37% accuracy, 96.34% precision, 96.37% recall, and a 96.35% F1-score**, consistently outperforming the single representation classifier. Error analysis via confusion matrix showed reduced misclassification across TILs categories, with OralTILs-ViT achieving the highest TOPSIS score and rank it is established that it is the best performing method. In conclusion, our study demonstrates that the proposed OralTILs-ViT i.e. combining weakly supervised cellular density map with tissue-level information significantly outperforms tissue-only approaches in TILs classification. Future work of this study may include expanding the dataset, testing on other cancers, and developing, an end-to-end system for real-world applications.

## Data Availability

The Panoptils dataset used in the study are available in https://sites.google.com/view/panoptils/ and TCGA-HNSCC available in https://portal.gdc.cancer.gov/.

## Code availability

The codes of the study will be publicly available at: https://github.com/Barun-Barua/OralTILs-ViT.git upon acceptance.
